# Bandgap tuning of mixed organic cation utilizing chemical vapor deposition process

**DOI:** 10.1038/srep37378

**Published:** 2016-11-22

**Authors:** Jeongmo Kim, Hyeong Pil Kim, Mohd Asri Mat Teridi, Abd. Rashid bin Mohd Yusoff, Jin Jang

**Affiliations:** 1 Advanced Display Research Center, Department of Information Display, Kyung Hee University, Dongdaemoon-gu, Seoul 130-701, South Korea; 2 Solar Energy Research Institute, Universiti Kebangsaan Malaysia, 43600 Bangi, Selangor, Malaysia

## Abstract

Bandgap tuning of a mixed organic cation perovskite is demonstrated via chemical vapor deposition process. The optical and electrical properties of the mixed organic cation perovskite can be manipulated by varying the growth time. A slight shift of the absorption band to shorter wavelengths is demonstrated with increasing growth time, which results in the increment of the current density. Hence, based on the optimized growth time, our device exhibits an efficiency of 15.86% with negligible current hysteresis.

Recently, a new type of solar cell technology has emerged, that promises low manufacturing costs, as well as stable, lightweight, and flexible systems compared to the silicon type solar cells. In the original design, the absorbing material is made up of hybrid organic-inorganic elements (CH_3_NH_3_PbI_3_) arranged in a perovskite structure. Since its introduction in 2009 by Kojima and co-workers^1^, its power conversion efficiency has leapfrogged from 3.8% to slightly above 21.2% by EPFL in 2015 and 22.1% by KRICT/UNIST in 2016^2^,^3^. The quick improvements were due to the simple deposition method required and it did not rely on the use of chemicals in limited supply, unstable solvents, and high temperature annealing. Furthermore, perovskites proved to be excellent absorbers with: suitable direct bandgaps, high absorption coefficients and excellent carrier transport^4^,^5^,^6^.

There are two commonly used techniques for producing high quality absorber layer perovskite solar cells, namely spin-coating and chemical vapor deposition (CVD). The spin-coating method has already been proven in term of performance, not only in perovskite solar cells, but in other photovoltaic devices such as organic solar cells, dye-sensitized solar cells and quantum dot solar cells. However, spin coating has a major problem for fabrication of large scale solar cells that still remains unsolved. The problem is in controlling the thickness and in obtaining a homogeneous film. This type of method results in a film with a non-uniform layer, with pinholes, which can reduce the device performance^7^,^8^,^9^. To overcome these problems, the CVD method is one of the solutions which can greatly impact the performance of perovskite solar cells. CVD is a promising method to produce planar films for fabricating the perovskite solar cells. The films obtained by CVD have already proven to exhibit improved crystallinity and have achieved suitably high charge carrier mobility, as well as preventing shunting and leakage currents under revers bias^10^. The process of CVD, which is almost similar to aerosol assisted chemical vapor deposition (AACVD), can be explained by the reaction of two or more gaseous chemicals created from volatile precursors that are transported to the reaction chamber, where the gaseous chemicals decompose on a heated substrate^11^. In CVD, uniform multi-component films with excellent reproducibility and precise control over the composition can be fabricated^12^. Additionally, films produced by CVD show better adhesion to the substrate compared to other methods. Furthermore, the homogeneity (size and structure) of the film can be easily controlled by controlling the chemical concentration, deposition time, substrate temperature and deposition rate^11^.

In this study, we demonstrate the combination of two different film deposition methods; spin-coating (electron and hole collector), and CVD with optimized growth time to deposit MA_X_FA_1−X_PbI_3_ thin films. The constructed perovskite device with the optimized fabrication procedure resulted in an energy conversion efficiency of up to 15.86%, which was stable, reproducible and suitable for large scale manufacturing.

## Results and Discussion

**Figure 1:**
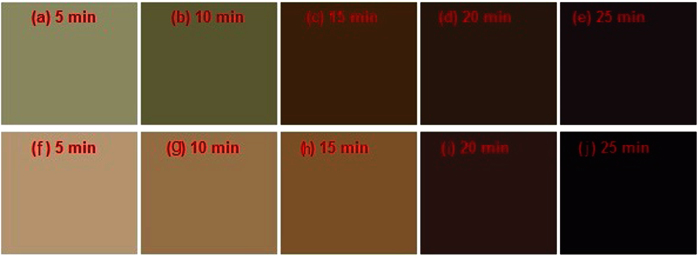
Photographs of MA_x_FA_1−x_PbI_3_ films with (**a**–**e**) 10 and (**f**–**j**) 400 sccm H_2_ flow rates with growth time increasing from 5 to 25 min.

A series of perovskite samples were prepared at 80 °C by CVD at two different flows of H_2_, (10, and 400 sccm) for different growth times of 5, 10, 15, 20, and 25 min. Figure 1 shows photograph images of the samples to illustrate the changes in the film’s color. The different colors of the deposition represent various concentrations of FAI and lead iodide. For a longer growth time of FAI, the film changes to form the perovskite, and turns from yellow to black as seen in Fig. 1. The color is dependent on the amount of FAI deposited because the yellow color belongs to the converted Pbl_3_. At a low H_2_ flow rate (Fig. 1a), the nucleation occurs for shorter growth durations. The nucleation and growth of the perovskite occurs only when a critical level of supersaturation is reached. At lower H_2_ flow rates and hence at lower partial pressure, this critical level of supersaturation is achieved only at longer growth durations.

As the H_2_ flow rate increases, this threshold of supersaturation is achieved at relatively shorter growth durations. Furthermore, our results also suggest that as the H_2_ flow rate increases, the density of the nuclei increases (Fig. 1b,e). At the slightly longer growth rate (Fig. 1b,g), the perovskite growth rate is visibly influenced by the H_2_ flow rate, which is clearly visible in the photograph images. Whereas when the H_2_ flow rate is very low (10 sccm), the nucleation and growth are reduced significantly, as compared in Fig. 1b,c,g,h. The grown perovskite films consist of irregular-shaped grains of different sizes at our CVD conditions (data not shown). We also observed that at a low H_2_ partial pressure, the perovskite domain features irregular shapes (data not shown). The rough, inhomogeneous, and irregular formation during perovskite growth influences its electrical properties. It is also worth noting that the growth time of the perovskite is relatively essential and must be carefully controlled to avoid any issues with over supersaturation (Fig. 1e,j).

**Figure 2:**
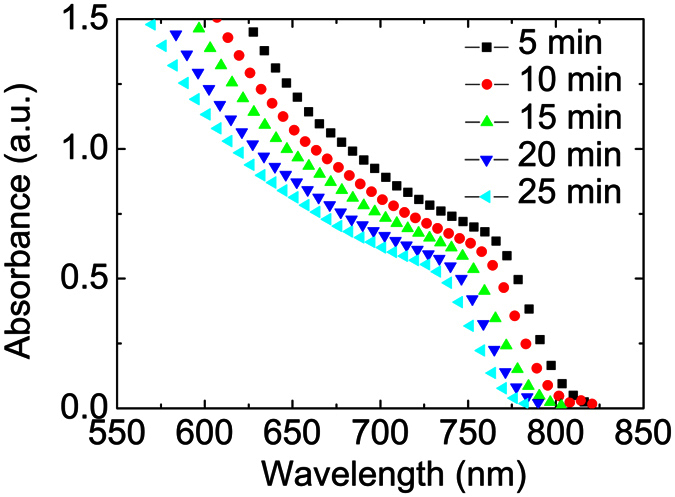
Absorption spectra of MA_x_FA_1−x_PbI_3_ films with 10 sccm H_2_ flow rates at different growth time from 5 to 25 min.

In order to determine the effect of the growth time, we varied the growth time at a fixed time interval of 5 min. The UV-Vis absorption spectra of the MA_x_FA_1−x_PbI_3_ perovskite films on ITO/ETL substrates are shown in Fig. 2. It can be seen that the onset of the absorption band becomes slightly blue-shifted with increased growth time; from 808 nm (1.54 eV) for 5 min to 770 nm (1.60 eV) for 25 min. The results corroborate with the observed color changes as seen in Fig. 1. The respective bandgaps of the MA_x_FA_1−x_PbI_3_ perovskite films are shown in Table 1.

**Figure 3:**
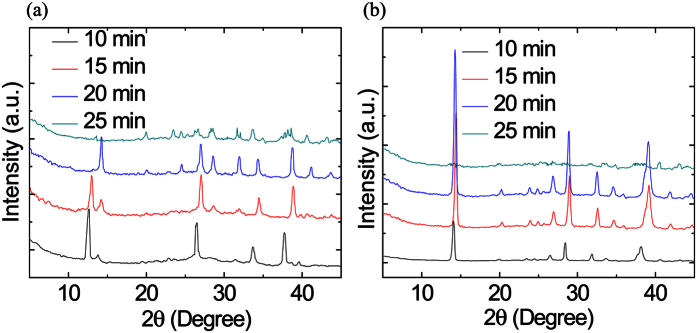
XRD patterns of MA_x_FA_1−x_PbI_3_ films with (**a**) 10 and (**b**) 400 sccm H_2_ flow rates with growth time increasing from 10 to 25 min.

X-ray diffraction spectroscopy measurements were performed in order to check the crystallinity of MA_x_FA_1−x_PbI_3_ with different H_2_ flows (10 to 400 sccm) at growth times of 5, 10, 15, 20, and 25 min. From the XRD patterns of PbI_2_ films, the main reflections of (001) at 12.62°, (002) at 25.4°, (003) at 38.54°, and (004) at 52.30° are in agreement with the previous reported work^12^,^13^,^14^,^15^. The FAI gives reflections at 14.07°, 28.36°, 31.82°, and 43.14°, which are assigned as the (110), (220), (310) and (330) planes, respectively, and it shows an orthorhombic crystal structure with preferred a orientation in the (110) direction^16^. As the amount of FAI was increased, the reflections that correspond to FAI also increased, but the reflections due to the lead iodide decreased until the color transitioned from yellow to black. In other words, after the reaction finished, all the identical peaks of MA disappeared, and many new reflections of MA_x_FA_1−x_PbI_3_ were observed, which means that the conversion from hexagonal MA into a mixed cation perovskite MA_x_FA_1−x_PbI_3_ is complete. Figure 3a shows the XRD patterns of the MA_x_FA_1−x_PbI_3_ structure, and it can be seen that the MA_x_FA_1−x_PbI_3_ structure becomes destroyed after 25 min of growth time. After further increasing the growth time to 25 min, all the main reflections disappeared (data not shown).

In Fig. 3b, after the films were grown at 400 sccm for 10 min, weak reflections of PbI_2_ at 12.62° and 38.54° begin to appear. With a prolonged growth time, the intensity of the reflections of PbI_2_ gradually increased, while the main reflections of FAI disappeared after growing for 25 min. The results showed that the amount of lead iodide increased for the films growth at 400 sccm but reduced the performance of the devices. From the photographs taken, there were apparent color changes among the samples. Generally, the annealing temperature in the solution route is 80 °C in a glove-box or in dry air conditions. MA_x_FA_1−x_PbI_3_ films with poor morphology quality will decompose quickly in ambient open air, especially under high humidity conditions.

**Figure 4:**
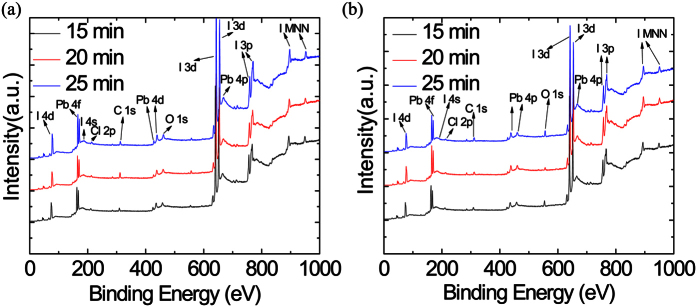
XPS spectra of MA_x_FA_1−x_PbI_3_ films with (**a**) 10, and (**b**) 400 sccm H_2_ flow rates with growth time increasing from 15 to 25 min.

In our study, the perovskite films are prepared under various growth times and H_2_ flow rates. The samples grown at low and high H_2_ flows show no apparent morphology difference except their colors. We started at a low H_2_ flow rate ranging from 5–25 min. XPS measurements were conducted to ensure that the 25 min growth time at low H_2_ sccm flow resulted in more FAI in the sample compared to that of a sample with 15 min growth time, as shown in Fig. 4. The amount of FAI used is crucial since the deposition process is not self-limiting, where the growth time of FAI must be monitored otherwise all the film will become supersaturated with FAI. In the end, the PSCs were assembled by sequentially spin-coating the hole transport materials (HTM), tungsten oxide nanoparticles (WO_3_) and by evaporating the Ag electrode.

**Figure 5:**
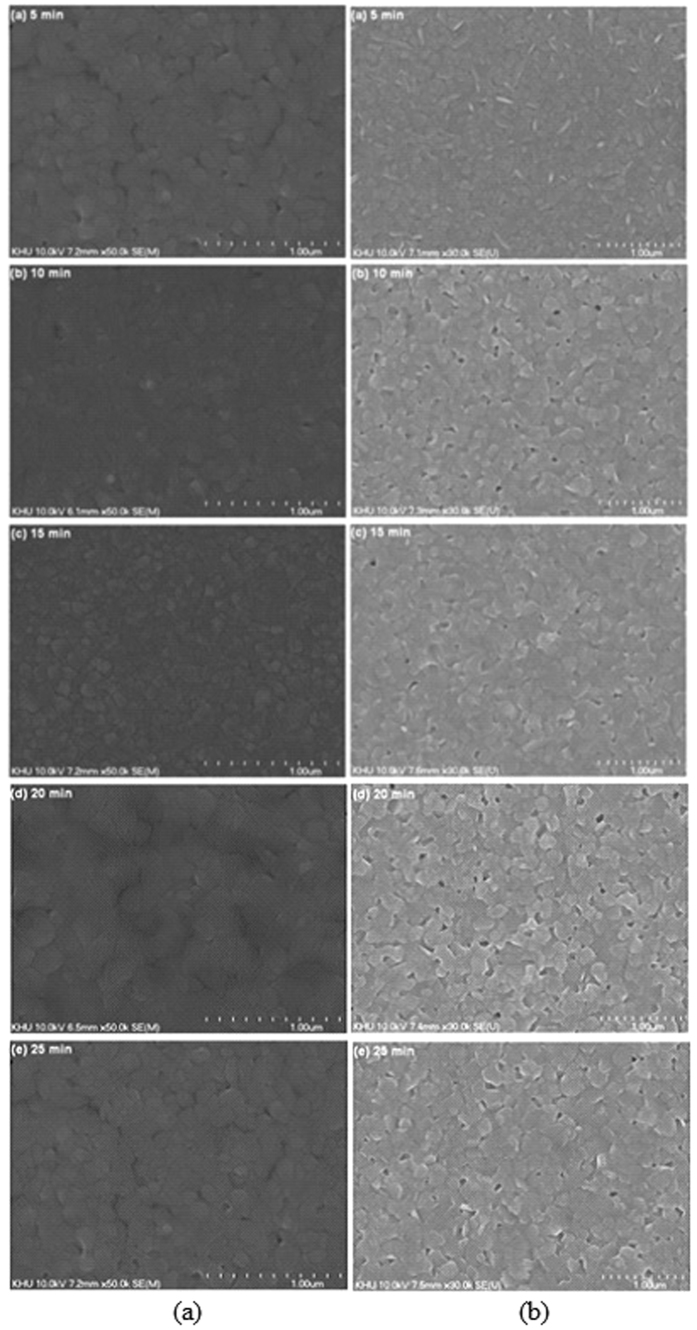
SEM images of MA_x_FA_1−x_PbI_3_ films with (**a**) 10, and (**b**) 400 sccm H_2_ flow rates with growth time increasing from 5 to 25 min.

In the CVD method, the quality of the perovskite film may depend on various factors such as temperature, gas flow rate and reaction time etc. SEM surface images of perovskite films are shown in Fig. 5. As expected, the morphology of perovskite films was significantly affected by the reaction time and gas flow rate. The film prepared for 15 min is ideal as it is very smooth and densely packed. There are no holes or crevices between the grains. This is the advantage of preparing perovskite films using CVD. The morphology of the perovskite film is an important factor that determines its photovoltaic performance in a complete cell. The perovskite film obtained at 10 sccm for 5 min is composed of a large number of small crystallites (size 50–500 nm) with several voids. On the other hand, the film obtained at 15 min is very dense with large crystallites. No voids and pinholes were observed even at a magnification of 100,000. The perovskite film obtained at 400 sccm for 15 min has less quality compared to that of 10 sccm for 15 min, several pinholes were observed on the surface. Further prolonging the processing time >15 min leads to less desirable features. In the case of 10 sccm for 20 and 25 min, although the perovskite films did not exhibit any pinholes, the films are less dense and less uniform. In contrast, in the case of 400 sccm at 20 and 25 min, the presence of pinholes or voids becomes more pronounced compared to the shorter times. This leads to poor photovoltaic performance.

We observed that the sizes of the grains become bigger when longer growth times are used. Moreover, the films become less compact with many pinholes. We can rationally assume that a shorter lifetime exists when more pinholes are in the film at the high H_2_ flow rate. We also assumed that the abundant surface area, pinholes, and defects in thin films should be responsible for the quenching. For the longer growth time, the boundary effect becomes less significant.

**Figure 6:**
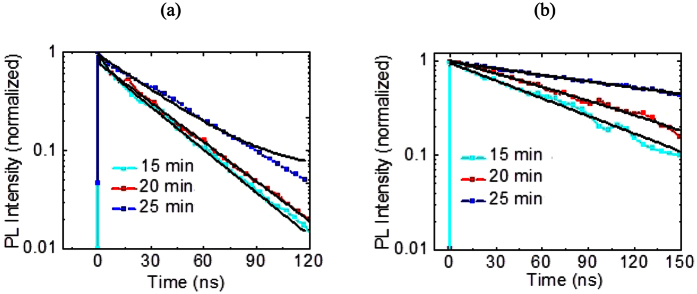
PL decay curves of MA_x_FA_1−x_PbI_3_ at different growth times from 5 to 25 min for (**a**) 10, and (**b**) 400 sccm H_2_ flow rates upon excitation at 517 nm, 90 nJ/cm^2^.

As shown in Fig. 6, the growth dependency demonstrated that the lifetime of ~63.22 ns was found for grains between 1 μm and 1.25 μm in size (Table 2). This shows that when the grain size becomes too big along with many pinholes, it increases the lifetime. For the lower flow rate of H_2_, the size of the grain becomes larger when the growth time becomes longer. Furthermore, the films also become less dense, less compact and inhomogeneous. For the growth time above 15 min, the boundary effect becomes less significant with apparent pinholes. Shorter growth times show dramatically reduced lifetime. This proves that when the boundary becomes insignificant, it demonstrates longer lifetime.

**Figure 7:**
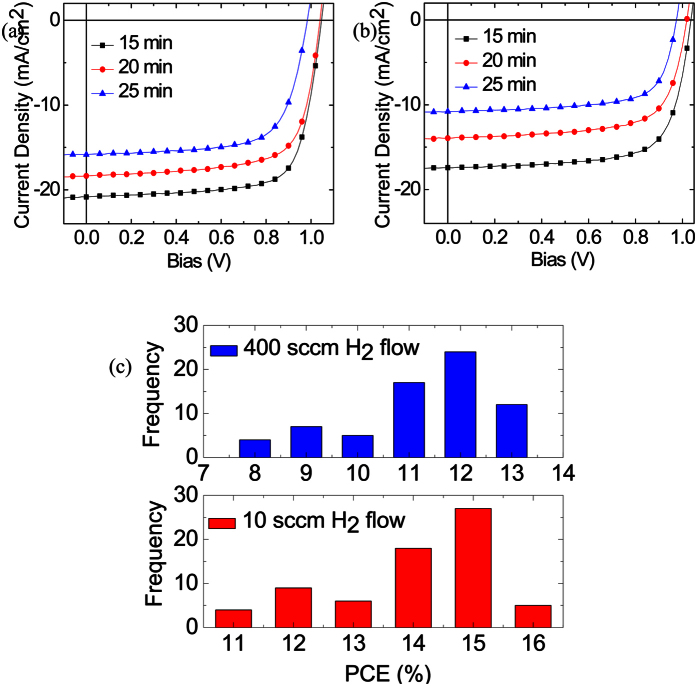
J-V characteristics of perovskite solar cell with (**a**) 10, (**b**) 400 sccm H_2_ flow rates with growth time increasing from 15 to 25 min under 100 mW/cm^2^ AM1.5 illumination and (**c**) the histogram of PCE for 138 devices prepared under 10 and 400 sccm H_2_ flows.

For comparison, perovskite films produced with low and high H_2_ flows under different growth rates were used to fabricate perovskite solar cells and their performance data was collected and is shown in Fig. 7, Tables 3 and 4. The J–V characteristics of the average of 63 PSCs are illustrated in Fig. 6a and b. PSCs with higher H_2_ flow, as well as longer growth rate, give an undesirable efficiency of 2.59% (data not shown), which is presumably due to a large amount of charge traps and the high carrier recombination in the perovskite absorber. In contrast, all PSCs fabricated with slow H_2_ flow rates (Fig. 7a) demonstrated higher efficiencies of above 10%, and exhibited good reproducibility. The PSCs prepared with low H_2_ flow give an average open-circuit voltage (V_OC_) of ~1.02 V, a short current density (J_SC_) ranging from 11–21 mA/cm^2^, and fill factor (FF) of ~70%. The best average device, as shown in Fig. 7a, is obtained from a growth time of 15 min, which provides average power conversion efficiency (PCE) of 15.86% with a V_OC_ of 1.04 V, J_SC_ of 20.85 mA/cm^2^, and FF of 73.15%. The enhanced efficiency was due to the high film quality, and the presence of FAI. Moreover, PSCs fabricated with a longer growth time (25 min) at a slow H_2_ flow still showed an efficiency of 10.78% with the V_OC_, J_SC_, and FF of 0.98 V, 15.85 mA/cm^2^, and 69.42%, respectively. This strongly verifies the stability and high quality of our perovskite materials. The small series resistance of the PSC (not shown) indicates that the interphase contact is good and the conductivity of every layer of the device is high. A high shunt resistance also suggests that the power loss in the device via an alternate current path is very small, resulting in a high FF. It is worth noting that shorter growth times for low and high H_2_ flow resulted in poor photovoltaic performance. We attribute these observations to the incomplete intercalation, which leads to inhomogeneous surface morphology as well as irregular-shaped grains (data not shown). In this study, 138 devices were fabricated. Figure 7c shows a histogram of the device performances for all cells. It indicates that the high PCE is a general result for the inverted mixed organic cation devices based on a perovskite film utilizing CVD.

Another important figure of merit in a perovskite solar cell is the stability and the hysteresis of the device. Unlike single-crystal silicon solar cells which have an average of 20-year lifetime, the lifetime of perovskite solar cell remains a challenge and is hotly debated. To date, only several fabrication techniques and architectures demonstrate promising lengthy lifetime^17^,^18^,^19^,^20^,^21^,^22^,^23^,^24^,^25^,^26^.

**Figure 8:**
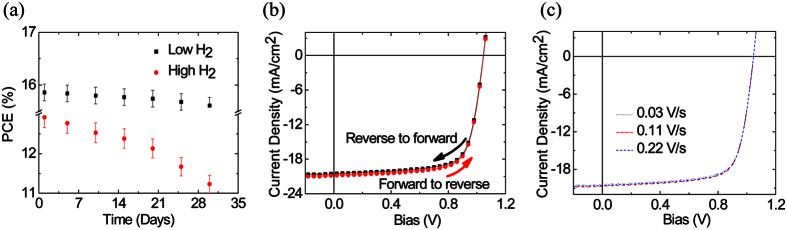
(**a**) Stability of perovskite solar cell under ambient conditions for perovskite solar cells fabricated at low and high H_2_ sccm flow rate for 15 min growth time. (**b**) J–V characteristics for the perovskite solar cell fabricated with low H_2_ sccm flow rate at 15 min growth time: (**b**) different scanning directions and (**c**) different voltage sweep rates.

Figure 8a illustrates the stability measurements of our perovskite solar cell measured 30 days after initial fabrication. Solar cells fabricated with low H_2_ flow demonstrated the best stability performance compared to that of the devices with higher H_2_ flow. This is probably due to higher iodide concentration in the film with shorter growth times.

Unlike devices utilizing chloride^27^, the stability in our fabricated devices seems to be better, thus, it looks like the halide component has an influential impact on stability. We believe there is still room for improvement in terms of cell lifetime before we can take it to the next level. In order to ensure the accuracy of our measurements, we extended our study in different scanning directions and at numerous voltage sweep rates. This is due to the fact that scanning directions, light soaking, voltage sweep rate and pre-conditioning of the device at a forward bias were all found to have a remarkable impact on the hysteresis^19^. As can be seen from Fig. 8b,c, the devices prepared in this study showed negligible current hysteresis, regardless of the various voltage sweep rates and/or scanning direction. This implies that our J-V characteristics are reliable. We attribute this behavior to the low surface defect densities of the perovskite films formed by the low H_2_ flow rate.

**Figure 9:**
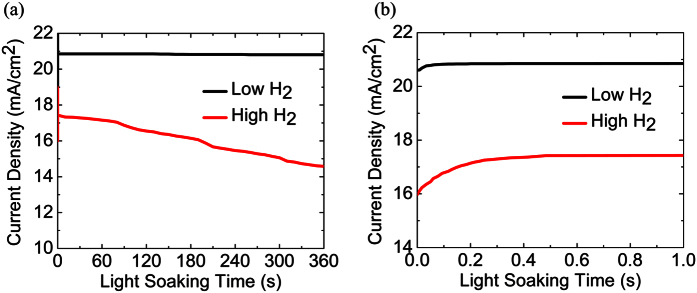
Current density variation of perovskite solar cells fabricated at low and high H_2_ sccm flow rate for 15 min growth time with light soaking time under applied bias voltage at maximum power for (**a**) 360 s and 1 s (at initial stage).

To further understand why our CVD perovskite solar cells demonstrated less current hysteresis compared to the previously reported work^28^, we evaluated the stabilization of current density for the film prepared at the 10 sccm H_2_ flow rate for 15 min as it showed some current hysteresis. Figure 9a,b show the variation of the current density at maximum power condition under optimum bias with the light soaking time under 1.5 illumination. The current density of the fabricated mixed-organic cation swiftly stabilized in <0.2 s and remained almost constant up to 360 s under continuous light illumination. It asserts that the mixed-organic cation MA_x_FA_1−x_PbI_3_ perovskite solar cells with WO_3_ exhibit less current hysteresis and better stability.

Moreover, the current density generated by mixed-organic cation MA_x_FA_1−x_PbI_3_ perovskite solar cell is defined as:


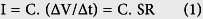


where I = current density, C = capacitance, ∆t = delay time, ∆V = voltage difference, and SR = scan rate.

When the photogenerated charge carriers in MA_x_FA_1−x_PbI_3_ being transported to the adjacent layer and there are no accumulated charge carriers in itself regardless to SR; thus direct current capacitance of MA_x_FA_1−x_PbI_3_ is completely independent on SR. Contrarily, when the photogenerated charge carriers by MA_x_FA_1−x_PbI_3_ are recombined or accumulated at traps in mesoscopic TiO_2_/MA_x_FA_1−x_PbI_3_/WO_3_, the J–V characteristics will eventually demonstrate current hysteresis with respect to both SR and scan direction due to the charging and discharging of internal capacitance-elements. Hence, the less current hysteresis for the perovskite solar cell fabricated with low H_2_ sccm flow rate (15 min growth time) is related with the fact that the charge accumulation at traps in mesoscopic TiO_2_/MA_x_FA_1−x_PbI_3_/WO_3_ is significantly reduced by optimum growth time and thereupon the flux of electrons and holes are balanced.

**Figure 10:**
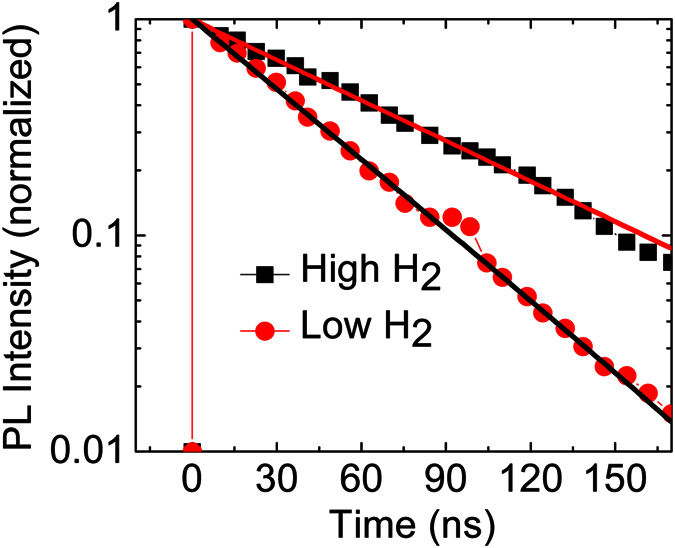
PL decay curves of TiO_2_/MA_x_FA_1−x_PbI_3_/WO_3_ of different H_2_ flow rates upon excitation at 517 nm, 90 nJ/cm^2^.

Assuming that the intrinsic properties of MA_x_FA_1−x_PbI_3_ are unchanged, the charge accumulation at TiO_2_/MA_x_FA_1−x_PbI_3_/WO_3_ is related to charge transfer at the mesoscopic TiO_2_/MA_x_FA_1−x_PbI_3_/WO_3_ interface and charge transport, as well. In order to evaluate the charge transfer or charge separation/injection behavior at the low H_2_ flow rate for 15 min growth time, we measured the transient PL (photo-luminescent) decay curves of mesoscopic MA_x_FA_1−x_PbI_3_ perovskite hybrid films (low and high flow rates) as displayed in Fig. 10. The transient PL spectra suggest that the charge carriers generated in MA_x_FA_1−x_PbI_3_ (at low H_2_ flow rates) are more efficiently transferred into the TiO_2_ mesoscopic electrode compared to of that of the high H_2_ flow rate. The transient PL spectrum were best fit by using three exponential functions defined as


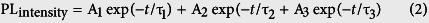


where A_1_, A_2_, and A_3_ are the amplitudes and τ_1_, τ_2_, and τ_3_ are the decay times, respectively. The decay time of low and high H_2_ flows were 1.93 ns and 2.46 ns, indicating that the charge transfer rate and the charge separation efficiency of mesoscopic MA_x_FA_1−x_PbI_3_ perovskite solar cells are enhanced by the low H_2_ flow rate. Other measurements have also confirmed the above analysis on MA_x_FA_1−x_PbI_3_ perovskite solar cells. These observations suggest that the PL decay of MA_x_FA_1−x_PbI_3_ occurs via three different temporal processes which are most probably associated to the morphological structure of the film. We conclude that the current hysteresis with respect to the scan direction can be also reduced by using a low H_2_ flow rate because the charge carriers generated in MA_x_FA_1−x_PbI_3_ are more effectively injected into TiO_2_ electrode; by this means balancing the flux of electrons and holes.

## Conclusions

In summary, we have seen that the H_2_ flow during the low pressure CVD had a significant effect not only on the physical properties, but also on the electrical properties of perovskite. Nucleation and grain growth of perovskite increased at higher hydrogen flows. Furthermore, more oxygen-related functional groups, like amorphous, probably contributed to defects or contamination of the perovskite surface at higher hydrogen flow rates. The perovskite film derived from this approach exhibits full surface coverage, uniform grain structure with grain size up to micrometers and 100% precursor transformation. A film evolution study on perovskite transformation indicates an appropriate rearrangement of FAI film during the intercalation of MA driven by the reduction of grain boundary energy. The negligible current hysteresis was attributed to the improved charge separation/injection from MA_x_FA_1−x_PbI_3_ into TiO_2_. Moreover, the current hysteresis might be eliminated by the reduced surface traps via low H_2_ flow rate since the capacitive elements might be diminished. Facilitated by the excellent film quality, the MA_x_FA_1−x_PbI_3_ materials enable an impressive device PCE of 15.86% in a planar architecture. CVD presents a simple, controllable, and versatile approach to the pursuit of high-quality perovskite films and the resulting high-performance PV devices.

### Experimental section

#### Substrate preparation

FTO glass (Argos Organic Chemicals, 7 Ω/sq.) was cleaned with detergent, rinsed with deionized water, and sonicated in 2-propanol. A compact layer of 65 nm TiO_2_ was then deposited via spin-coating using a precursor solution of acetylacetone, Ti (IV) isopropoxide, and anhydrous ethanol (3:3:2) on a pre-heated hot plate at 480 °C.

#### Perovskite film growth by CVD

A compact PbI_2_ layer was first deposited by spin-coating the PbI_2_ precursor in DMF (400 mg/mL) onto 65 nm of TiO_2_. Then the deposited substrates were placed in a tube-furnace where FAI was first deposited onto substrates, which was then removed for thermal annealing at various temperatures to form a FA_0.4_PbI_2.4_ intermediate complex. The substrates were placed back in the tube furnace, where the MAI was deposited onto FA_0.4_PbI_2.4_ film. The stack of FA_0.4_PbI_2.4_/MAI film was later removed for thermal annealing at 100 °C for 2 h to crystallize the MA_x_FA_1−x_PbI_3_. Thickness was monitored with a quartz-crystal microbalance and additionally measured using a profilometer. Throughout this experiment, the furnace was pumped down to 100 Pa under at various flow rates of H_2_ gas. The perovskite layers were optimized by varying the H_2_ gas flow rate (10 sccm and 400 sccm), temperature, deposition time (5 min to 25 min) and the amount of precursors.

*Dimensions of horizontal reactor glass tube*[Table t5].

*Dimension of furnace structure*.[Table t6]

*Dimension of heating systems*.[Table t7]

#### Device fabrication

The typical device configuration was FTO/TiO_2_/MA_x_FA_1−x_PbI_3_/WO_3_/Ag, where the hole transport layer, tungsten oxide nanoparticles (Argos Organic Chemicals) was spin coated at 1000 rpm for 30 s followed by 80 °C annealing treatment for 10 min. Top electrode was silver, deposited by thermal evaporation (~1.0 × 10^–7^ Torr) through a shadow mask. The active area was 0.2 cm^2^.

#### Solar cell measurements

The performance of the perovskite solar cells was obtained from J-V characteristics measured using a Keithley 2400 LV source meter. Solar cell performance was measured using a solar simulator, with an Air Mass 1.5 Global (AM 1.5 G) and had an irradiation intensity of 100 mW/cm^2^. All measurements were carried out at room temperature, under a relative humidity of 60%. The EQE measurements were performed using the EQE system (Model 74000) obtained from Newport Oriel Instruments USA and HAMAMATSU calibrated silicon cell photodiodes as a reference diode. The wavelength was controlled with a monochromator of 200–1600 nm.

### Film Characterization

The X-ray diffraction (XRD) analysis was performed using a XRD Diffractometer X’Pert PRO with Cu Kα target (λ = 0.154 nm) at a scan rate of 2°/min and an operating voltage of 40 kV with a current of 100 mA. Scanning electron microscope images were performed using a HITACHI S-4700. X-ray photoelectron spectroscopy (XPS) (Multilab. ESCA 2000) in KOPTRI. The film thickness was measured by Dektak AlphaStep Profiler. Time-resolved photoluminescence (TRPL) measurements were carried out using Edinburgh Instruments Ltd. FLSP920 with a 465.8 nm pulsed diode laser excitation source with ~100 ps pulse width and a laser irradiance of ~40 μW/cm^2^.

## Additional Information

**How to cite this article**: Kim, J. *et al*. Bandgap tuning of mixed organic cation utilizing chemical vapor deposition process. *Sci. Rep*. **6**, 37378; doi: 10.1038/srep37378 (2016).

**Publisher’s note:** Springer Nature remains neutral with regard to jurisdictional claims in published maps and institutional affiliations.

## Figures and Tables

**Table 1:** The calculated bandgap of MA_x_FA_1−x_PbI_3_ under 10 sccm H_2_ flow rate at various growth time from 5 until 25 min.

Time (min)	Calculated bandgap (eV)
5	1.54 eV
10	1.55 eV
15	1.57 eV
20	1.58 eV
25	1.60 eV

**Table 2:** PL lifetimes of glass/MA_x_FA_1−x_PbI_3_/WO_3_ films with (a) 10, and (b) 400 sccm H_2_ flow rates with growth time increasing from 15 to 25 min.

Growth time (min)	A*	T*	A^+^	T^+^
15	0.20	6.02	0.28	63.22
20	1.26	7.41	0.88	69.69
25	2.46	29.87	1.27	165.08

^*^For 10 sccm H_2_ and ^+^for 400 sccm H_2_ flow rates.

**Table 3:** The photovoltaic parameters of MA_x_FA_1−x_PbI_3_/WO_3_ solar cells under 10 sccm H_2_ flow rate with growth time increasing from 15 to 25 min under 100 mW/cm^2^ AM1.5 illumination.

Time (min)	J_SC_ (mA/cm^2^)	V_OC_ (V)	FF (%)	PCE (%)
5	13.44	0.83	56.23	6.27
10	17.85	0.99	68.55	12.11
15	20.85	1.04	73.15	15.86
20	18.38	1.04	70.57	13.49
25	15.85	0.98	69.42	10.78

**Table 4:** The photovoltaic parameters of MA_x_FA_1−x_PbI_3_/WO_3_ solar cells under 400 sccm H_2_ flow rate with growth time increasing from 15 to 25 min under 100 mW/cm^2^ AM1.5 illumination.

Time (min)	J_SC_ (mA/cm^2^)	V_OC_ (V)	FF (%)	PCE (%)
5	10.44	0.97	50.33	5.10
10	13.67	1.01	67.93	9.38
15	17.43	1.04	71.30	12.92
20	11.83	1.02	71.12	10.84
25	10.80	0.98	69.53	7.36

**Table 5 t5:** 

Component	Dimension (mm)
OD	45 ± 1
ID	40 ± 1
Length	1280 ± 1
Materials	Glass

**Table 6 t6:** 

Component	Dimension (mm)
Length	1000 ± 10
ID	50 ± 1
OD	55 ± 1
Operating temperature	1000 °C
Hollow tube materials	High temperature moulded ceramic
Construction materials	Heavy 2 mm metal fab sheets square shape
Support structure	4 pole for table top mounting
Design	3 heating zone type

**Table 7 t7:** 

Component	Dimension (mm)
Power control	Single phase with through the phase angle controlled drive
Heating elements	Designed to withstand heat up to 1100 °C
Type of elements	Solid type/Coil type Kanthal Al heating element
Total length of elements	1000 ± 10
Resistance of elements	1.24 Ω
Working temperature	1100 °C
Power	10 kW line load
Electrical line details	40 Amp
Power cable	4 core armoured cable with wire 2 m each
